# Melatonin upregulates BMAL1 to attenuate chronic sleep deprivation‐related cognitive impairment by alleviating oxidative stress

**DOI:** 10.1002/brb3.2836

**Published:** 2022-12-23

**Authors:** Yujie Hu, Jierong Yin, Guoshuai Yang

**Affiliations:** ^1^ Department of Neurology Central South University Xiangya School of Medicine Affiliated Haikou Hospital Haikou China

**Keywords:** BMAL1, melatonin, oxidative stress, sleep deprivation

## Abstract

**Purpose:**

To investigate the mechanism underlying the regulatory effect of melatonin on chronic sleep deprivation‐related cognitive impairment.

**Methods:**

Chronic sleep deprivation (CSD) model was established using the MMPM method. After the model was established, melatonin receptor agonist and inhibitor were given, respectively. Water maze was conducted to record the escape latency and the duration of crossing the platform of space exploration. The concentration of TNF‐α, IL‐6, MDA, and SOD was measured by ELISA. Immunofluorescence was used to determine the expression level of CD86 and CD206, while the mRNA expression of Bax, Bcl‐2, P65, IκB, and BMAL1 was detected by qPCR. Western blotting assay was utilized to determine the protein expression of Bax, Bcl‐2, P65, p‐P65, IκB, p‐I κB, and BMAL1.

**Results:**

Compared with the control, the escape latency was greatly increased on the second and third day, accompanied by the increased expression of TNF‐α, IL‐6, MDA, and SOD in serum. Furthermore, dramatically upregulated Bax, Bcl‐2, P65, IκB, and CD86 were observed in the model group, accompanied by the declined expression level of BMAL1 and CD206. Compared with the model group, the escape latency was declined, the concentration of TNF‐α, IL‐6, MDA, and SOD was decreased, the expression level of Bax, Bcl‐2, P65, IκB, and CD86 was declined, and the level of BMAL1 and CD206 was promoted by the treatment of the melatonin agonist, while the opposite results were observed under the treatment of the melatonin inhibitor.

**Conclusion:**

Melatonin upregulates BMAL1 to attenuate chronic sleep deprivation‐related cognitive impairment by alleviating oxidative stress.

## Graphical abstract

The present study aims to investigate the effect of melatonin on cognitive dysfunction in sleep‐deprived rats and the underlying mechanism. We speculated that melatonin upregulated BMAL1 to attenuate chronic sleep deprivation related cognitive impairment by allevaiting oxidative stress.

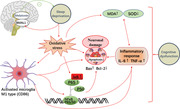

## 1 INTRODUCTION

Sleep deprivation (SD) is a pathological state in which the body is continuously awake and neither the body nor the energy level can be restored promptly (Yvan et al., 2017). As a result, SD makes it difficult or impossible to maintain a normal level of cognition and the ability to think effectively in daily life (Lo June et al., 2016). SD also activates oxidative stress in the hippocampus of the brain, further activating microglia and leading to neuroinflammation (Tahir et al., 2019). In addition, excessive autophagy and apoptosis of neurons will be triggered by SD, which ultimately leads to cognitive impairment and a series of psychiatric disorders (Yin et al., 2019). Therefore, it is urgent to find a new approach to alleviate sleep deprivation‐related cognitive impairment.

Sleep is controlled by circadian rhythms and homeostatic mechanisms. Circadian rhythm is a type of rhythmic activity with a period close to the circadian 24‐h period. The nuclear genes CLOCK and BMAL1 are upregulated in the absence of light stimulation during the night, leading to an increase in the levels of the corresponding CLOCK proteins and BMAL1 proteins, which further cause high PER and CRY protein levels in the cytosol; PER/CRY heterodimers enter the nucleus after reaching a certain threshold and negatively feedback to inhibit the level of the CLOCK/BMAL1 complex (Dunlap, 1999). BMAL1 is the only single clock gene deletion that contributes to complete ablation of all rhythms. BMAL1 downregulation aggravates atherosclerosis by encouraging oxidative stress (Xie et al., 2020). Deletion of BMAL1 will lead to the excessive prooxidant and proinflammatory phenotype (Early et al., 2018). Therefore, BMAL1 appears to be a potent regulator of oxidative stress and inflammation at the cellular and organismal levels.

Melatonin is an endocrine hormone secreted primarily by the pineal gland at night, which provides a variety of biological functions in the human circadian rhythm, including sleep and immune response (Rüdiger et al., 2012). Clinical trials have demonstrated that exogenous melatonin is effective for treating several types of sleep disorders, including circadian rhythm sleep‐wake disorder, insomnia, and parasomnia (Auger et al., 2015; Auld et al., 2017). However, the effects of melatonin on cognition remain unclear. Melatonin is an effective free radical scavenger with antioxidant, anti‐inflammatory, and anti‐apoptotic properties (Annia et al., 2013; Mauriz et al., 2013). Melatonin alleviates oxidative stress through a variety of mechanisms of scavenging ROS, and protecting glutathione peroxidase (GSH‐PX), catalase (CAT), and other important proteins and enzymes from oxidative damage. Studies have shown that melatonin has neuroprotective effects in progressive neurodegenerative diseases. However, few reports claimed the role of melatonin in cognitive impairment associated with SD. Therefore, the present study aims to investigate the effect of melatonin on cognitive dysfunction in sleep‐deprived rats and the underlying mechanism.

## 2 MATERIAL AND METHODS

### 2.1 Ethics statement

All experimental procedures were performed under the Institutional Guidelines of Care and Use of Animals and approved by the Animals Ethics Committee of Haikou Affiliated Hospital of Central South University Xiangya School of Medicine. Efforts were made to minimize animals use and suffering.

### 2.2 Animals, modeling, and grouping

24 Male 250–300 g SD rats were purchased from Sipford Biotechnology Co., Ltd (Beijing, China). Four groups were randomly and averagely divided in the present study (*n* = 6): Control (CON), Chronic sleep deprivation(CSD), CSD+melatonin agonist (agonist), and CSD+melatonin inhibitor (inhibitor). For the CSD modeling, there were 15 platforms in the 75.5 cm × 55.5 m × 45 m sleep deprivation box, with a diameter of 5.5 cm and a height of 8 cm. Before modeling, water was added into the tank with the water level 1 cm lower than the platform and the water was changed every day. Water and food were placed at the 15 cm cage cover on the platform. Two days before modeling, the rats were placed in a sleep deprivation box for 1 h per day. After the formal experiment initiated, the rats were placed in a sleep deprivation box for 6 h at the same time every day (10:00–16:00) to prevent them from entering the REM sleep state. During the 7 days of experiments, the rats were kept alternating between 12 h of light and 12 h of darkness per day. Animals in the agonist group were intraperitoneally injected with 10 mg/kg remelteon per day for 7 days, while the rats in the inhibitor group were intraperitoneally injected with 30 mg/kg N‐acetyl‐2‐benzyltryptamine per day for 7 days. Animals in the CON and CSD groups were intraperitoneally administered with 10 mL/kg normal saline per day for 7 days.

### 2.3 Water maze test

The device was equipped with a circular pool with a diameter of 1.5 m and a height of 0.5 m, which was artificially divided into four quadrants. One quadrant was equipped with a safety platform in the center. Water mazes were divided into positioning navigation experiments and space exploration trials. On the first day of the positioning navigation experiment, the water level was adjusted to 1 cm lower than the platform before placing the rats into the pool for free swimming. If the platform was reached by the rats, the escape latency was recorded as 60 s. The rats will be manually guided to the safe platform if they do not reach the platform for more than 90 s. The incubation period of each animal per day was determined as the average of the escape latency after entering the water in the four directions. From the second to the fifth day, the water level was 1 cm higher than the platform, and the rats were placed into the pool from four different directions. The swimming routes and escape latency of the rats were recorded. On the sixth day, the safety platform was removed and the rats were placed in the diagonal direction of the original safety platform. The duration of crossing the area of the original platform was recorded.

### 2.4 ELISA assay

The concentration of TNF‐α, IL‐6, MDA, and SOD in the serum was determined using the ELISA assay (MEIMIAN, Jiangsu, China). The serum was obtained following centrifugation, which was seeded in the 96‐well plate. After incubation, the solution was discarded to introduce conjugate reagents into the wells, followed by incubation at 37°C for 60 min. Subsequently, the wells were added with TMB and incubated for 15 min, followed by introducing the stop solution. Last, the microplate reader (Molecular Devices, Shenzhen, China) was utilized to obtain the OD value at 450 nm.

### 2.5 Nissl staining

The hippocampus tissues of each animal were collected and fixed in 4% paraformaldehyde, followed by gradient dehydration using different concentrations of ethanol solution. After paraffin embedment, the tissues were cut into 4–6 μm sections, which were then dewaxed and hydrated. Subsequently, the slides were dyed in methylene blue staining solution for 10 min, followed by addition of Nissl differentiation solution and stained for 3–5 min. The slides were then rinsed quickly with distilled water to prevent decolorization, dehydrated with anhydrous ethanol, incubated with xylene, sealed with neutral gum sealing, and lastly observed under the inverted microscope (OLYMPUS, Tokyo, Japan).

### 2.6 Immunofluorescence

After paraffin section dewaxing pretreatment, the section was put into the repair box and the antigen repair solution was added. The section was heated in the pressure pot until automatically deflated. After washing with PBS, the section was cooled for 2 min, and the antigen repair solution was discarded. The section was placed at the room temperature for 20 min, followed by immersion in PBS three times. Then, 5% BSA was added to the slides for 30 min at 37°C, and the fluid around the tissues was sucked up with absorbent paper, followed by addition of primary antibody against Iba1 (1:100) and placing in a wet box for incubation at 4°C overnight. After the slides were rewarmed to room temperature on the next day, the slides were immersed in PBS three times, and then diluted fluorescent secondary antibody Cy3 (1:200) was added. Subsequently, the slides were incubated with 5% BSA and sealed at 37°C for 30 min, followed by addition of the primary antibody against CD86 (1:200) and CD206 (1:200) and incubated at 37°C for 3 h. After the slides were rewarmed to room temperature, they were immersed in PBS for three times and then incubated with diluted fluorescent IgG/488 (1:100) antibody at 37°C for 30 min. Last, images were taken under fluorescence microscopy.

### 2.7 Real‐time PCR

RNAs were extracted from hDPCs using TRIzol reagent following different treatments, which were then transcribed to cDNA using a 1st‐Strand HiScript II Q RT SuperMix for qPCR (+gDNA wiper) Kit (Vazyme, China). The ChamQ Universal SYBR qPCR Master Mix (Vazyme, China) was used for PCR reaction. The 2^−ΔΔCt^ method was used for calculation of the gene levels. The primer sequences were shown in Table 1.

**TABLE 1:** Primer sequences

Genes	Sequences (5′−3′)
β‐actin F	GCCATGTACGTAGCCATCCA
β‐actin R	GAACCGCTCATTGCCGATAG
Bax F	GCGATGAACTGGACAACAAC
Bax R	GCAAAGTAGAAAAGGGCAACC
Bcl‐2 F	GCGTCAACAGGGAGATGTCA
Bcl‐2 R	TTCCACAAAGGCATCCCAGC
P65 F	GCAAAAGGACCTACGAGACC
P65 R	CGGGAAGGCACAGCAATA
Iκ B F	TGTCTACACTTAGCCTCTATCCAT
Iκ B R	GGGCAACTCATCTTCCGT
BMAL1 F	GACTCCAGACATTCCTTCCGC
BMAL1 R	CTATGTGGGGGTTCTCACCAAGAA

### 2.8 Western blot analysis

The cells were isolated with lysis buffer, which was further quantified with the BCA method (Elabscience, USA). The 12% SDS‐PAGE was used to separate proteins, followed by transferring them to the PVDF membrane (GE Healthcare Life, USA). After blocking, primary antibodies against Bax (Servicebio, China), P65 (Affinity, USA), p‐P65 (Affinity, USA), Iκ B (Affinity, USA), p‐Iκ B (Affinity, USA), BMAL1 (Affinity, USA), Bcl‐2 (Affinity, USA), and β‐actin (SolelyBio, China) were added and incubated at 4°C for 12 h, followed by the secondary antibody (CST, USA) for 90 min. Last, the level of proteins was quantified by analyzing the bands with the Image J software.

### 2.9 Statistical analysis

The data were expressed as mean ± SD, which were analyzed using one‐way ANOVA followed by the Tukey test. Repeated measurement data analysis of variance was used to analyze the results of escape latency in the Morris water maze. Statistically significant was determined when the value of *p* was lower than 0.05.

## 3 RESULTS

### 3.1 Chronic sleep deprivation impairs spatial learning in rats, and melatonin treatment reduces the extent of this impairment

**FIGURE 1:**
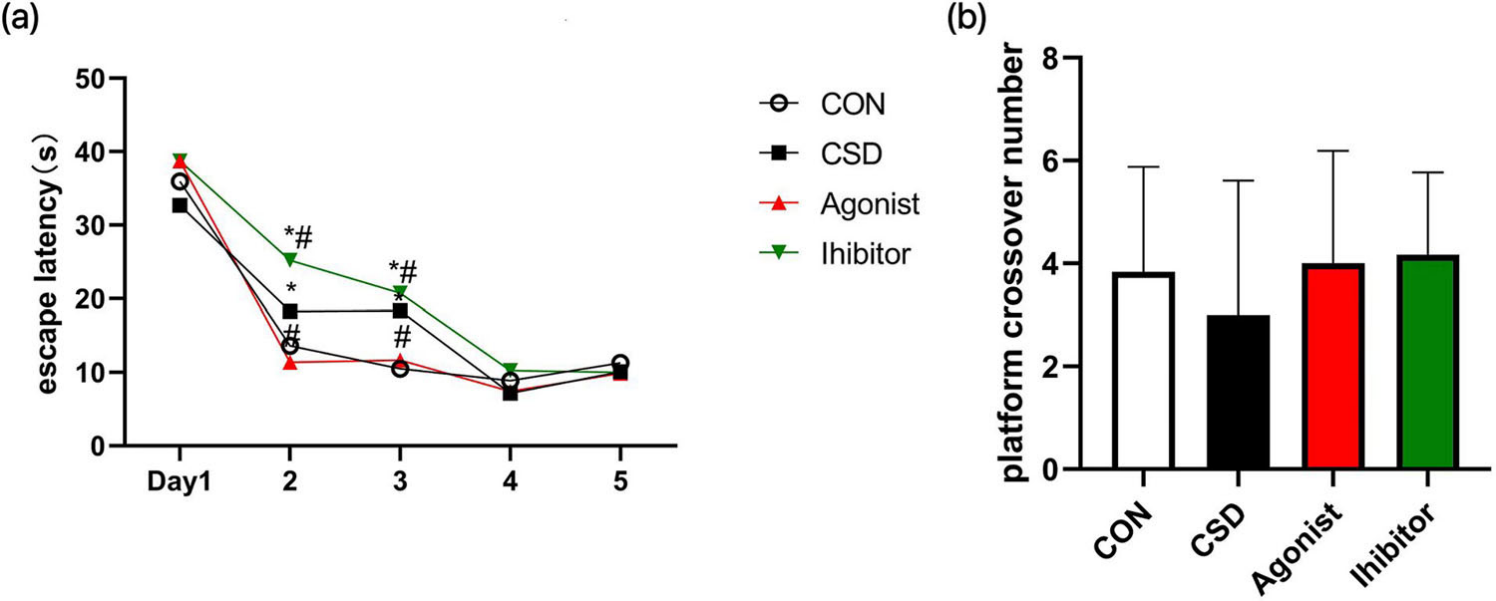
The results of the water maze test. **(a)The escape latency**. Compare to CON, the escape latency was greatly increased in the CSD group, which was significantly decreased by the treatment of melatonin agonist and highly changed by the treatment of melatonin inhibitor on the second and third day (P ＜ 0.05). **(b)The number of crossing the orignal platform**. There was no significant difference between the groups in the number of crossing the original platform. **Note**: *indicates a highly significant difference (P ＜ 0.05) between the normal control group; #indicates a significant difference (P ＜ 0.05) between the model group

Sleep is essential for maintaining good physical and mental health and normal cognition, and the sleep cycle is divided into REM sleep and NREM sleep, which may play important roles in different ways. NREM sleep may play an important metabolic role in correcting energy and nutritional imbalances after prolonged wakefulness, whereas REM sleep is more important in providing support for endogenous stimulation, neurogenesis, neurological and emotional development, and learning and memory formation (Jan et al., 2010). In the present study, we observed a significant increase in escape latency in the CSD group compared to the CON group, especially on the second and third days; the administration of melatonin agonists and inhibitors to CSD rats resulted in a significant decrease and increase in escape latency respectively (Figure 1a). Escape latency refers to the time from entering the water to climbing onto the cryptic escape platform and reflects the learning ability of the rats to find the exact location of the platform. The number of crossing of the original platform area is a key indicator to assess the memory ability of the rats, but there was no significant difference between the groups (Figure 1b). Thus, our results suggest that chronic sleep deprivation impairs spatial learning ability in rats, but the degree of impairment on memory ability is not significant, and the administration of melatonin agonist treatment reduces the impairment of chronic sleep deprivation‐induced spatial learning ability.

### 3.2 Chronic sleep deprivation downregulates BMAL1 expression and induces excessive oxidative stress, while melatonin treatment reverses this change

**FIGURE 2:**
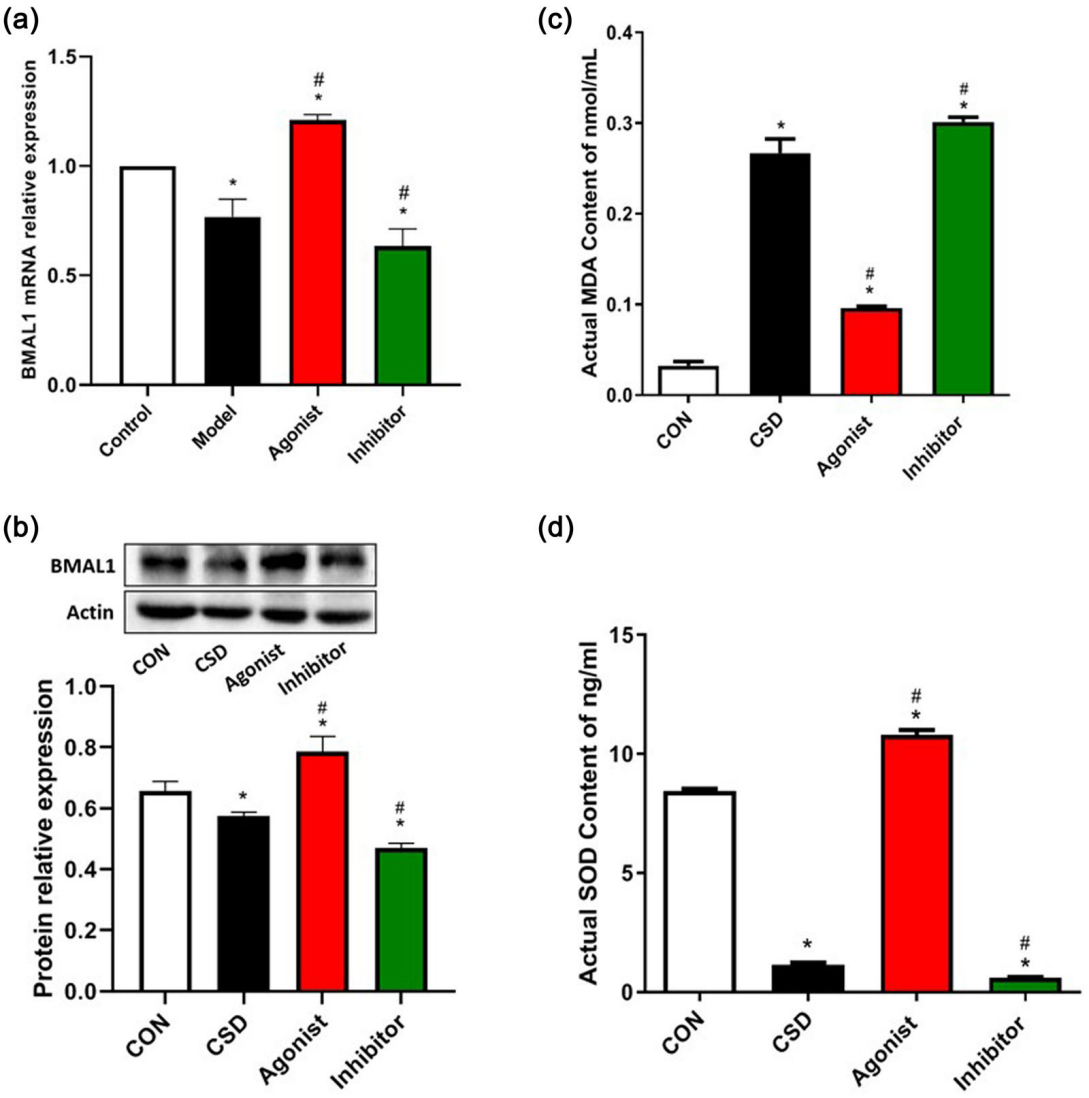
The PCR analysis and the Western blotting of BMAL1 and ELISA for SOD and MDA levels. **(a.b)The qPCR analysis and the Western blotting of BMAL1**. Compared to CON, the mRNA and protein level of BMAL1 dramatically declined in the CSD group (P ＜ 0.05). Compared to the CSD group, BMAL1 was upregulated by the treatment of melatonin agonist, but further declined by the treatment of melatonin inhibitor (P ＜ 0.05). **(c.d)ELISA for SOD and MDA levels**. Compared with CON, the levels of MDA increased significantly and the levels of SOD decreased significantly in CSD rats; which were greatly reversed by the treatment of melatonin agonist and aggravated by the treatment of melatonin inhibitor (P ＜ 0.05). **Note**: * indicates a highly significant difference (P ＜ 0.05) between the normal control group; #indicates a significant difference (P ＜ 0.05) between the model group

Conditions such as sleep deprivation or pulsed light exposure can disrupt circadian rhythm oscillations, thereby affecting the expression or transcriptional activity of clock genes, which in turn leads to a variety of pathologies, including accelerated aging, dementia, and vascular disease (Musiek et al., 2013), and clock genes including BMAL1, CLOCK, and retinoic acid‐related orphan receptor alpha (RORα) are involved in the regulation of immune and inflammatory cell function. It has been shown that BMAL1 knockout mice exhibit loss of behavioral and physiological circadian rhythms and show signs of increased systemic oxidative stress and accelerated aging (Wang et al., 2020). We found that BMAL1 gene and protein expression was down‐regulated in CSD rats, and its expression was lower after administration of melatonin inhibitors, but BMAL1 expression was significantly higher after administration of melatonin agonists compared to the previous two (Figure 2a,b). It indicates that the circadian rhythm of organisms is regulated by a complex feedback control loop consisting of biological clock genes within the suprachiasmatic nucleus, and there is a feedback regulation between melatonin, as one of the neurotransmitters and neuromodulators involved in the circadian rhythm, and the expression of clock genes.

In addition, to investigate whether CSD induces excessive oxidative stress, we measured the levels of SOD and MDA in serum which are very important parameters reflecting oxidative stress. MDA is a product of lipid peroxidation of polyunsaturated fatty acids by ROS, which reacts with various molecules (e.g., proteins, nucleosides, DNA, etc.) and may produce many adducts that promote cellular damage, and even lead to DNA mutation and induction of apoptosis (Lasisi et al., 2021). SOD is a key antioxidant enzyme that scavenges excess ROS and MDA, and its activity indirectly reflects the ability to scavenge oxygen radicals, and its reduced activity indicates antioxidant barrier damage. In the present study, we observed a significant increase in MDA levels and a significant decrease in SOD antioxidant levels in the CSD group compared to the CON group (Figure 2c,d), indicating that chronic sleep deprivation induces excessive oxidative stress in rats. However, the changes in serum SOD and MDA levels were reversed after melatonin supplementation treatment, resulting in an improvement of chronic sleep deprivation‐induced excessive oxidative stress.

### 3.3 Chronic sleep deprivation promotes the conversion of microglia to the M1 type, which increases the release of inflammatory factors, while melatonin receptor agonists can partially counteract neuroinflammatory damage

**FIGURE 3:**
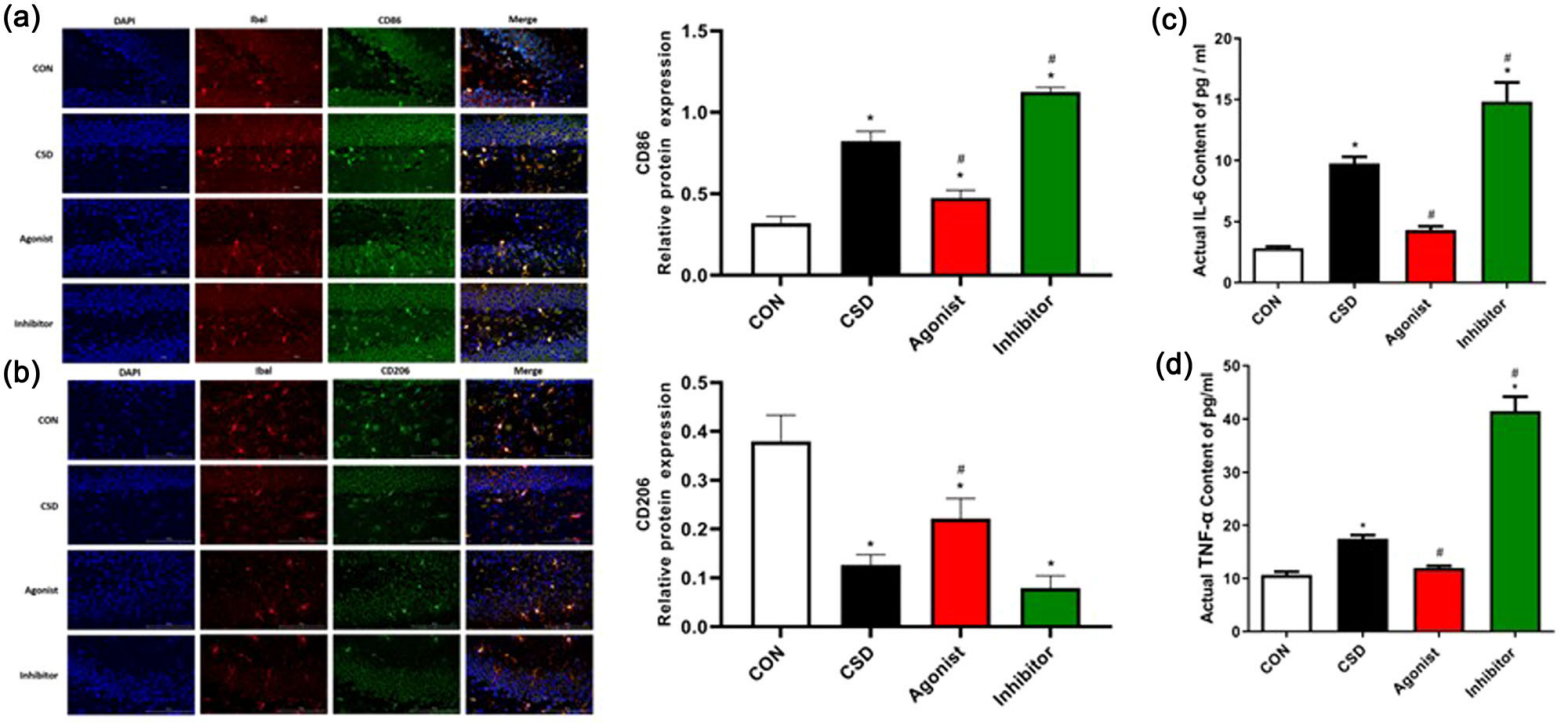
Analysis of immunofluorescence double‐staining results and ELISA for IL‐6 and TNF‐α levels. **(a.b)Analysis of immunofluorescence double‐staining results(400X)**. To investigate the relationship between microglia activation and M1/M2 type polarization in the sleep deprivation model, immunofluorescence double‐staining was used to detect the co‐localized expression of Iba1/CD86 and Iba1/CD206 in brain tissue. Compared with the control group, CD86 expression was significantly higher and CD206 expression was decreased in the model group, and the differences were statistically significant (P ＜ 0.05). Compared with the model group, CD86 expression was significantly decreased and CD206 expression was increased in the agonist group, and the difference was statistically significant (P ＜ 0.05); CD86 expression was significantly increased and the difference was statistically significant (P ＜ 0.05) in the inhibitor group, and CD206 expression was slightly decreased, but there was no significant difference. **(c.d)ELISA for IL‐6 and TNF‐α levels**. Compared to CON, significantly increased levels of IL‐6 and TNF‐α in CSD rats, which were greatly reversed by the treatment of melatonin agonist and aggravated by the treatment of melatonin inhibitor in CSD rats, which were greatly reversed by the treatment of melatonin agonist and aggravated by the treatment of melatonin inhibitor (P ＜ 0.05). **Note**: *indicates a highly significant difference (P ＜ 0.05) between the normal control group; #indicates a significant difference (P ＜ 0.05) between the model group

Microglia and their cytokines regulate neuronal activity during the normal sleep–wake cycle and in response to sleep deprivation, and microglia can respond to acute or recurrent chronic sleep loss through changes in the morphology and the levels of their regulated mRNAs or protein (Deurveilher et al., 2021). Under normal physiological conditions, microglia maintain a resting phenotype (M0 type) and are involved in the neuronal activity and maintaining homeostasis of the brain environment. However, in face of oxidative stress injury, such as that produced by chronic sleep deprivation, microglia are induced to a neurotoxic M1 phenotype that secretes pro‐inflammatory cytokines, chemokines, and reactive oxidants, and causes a neuroinflammatory response, impairs neurogenesis, and thus affects cognitive function. The microglia M2 phenotype, on the other hand, is an alternative activating phenotype that plays a role in neuroprotection, releasing anti‐inflammatory cytokines and preventing neuronal damage (Zhang et al., 2018). Therefore, we observed the expression of M1‐ and M2‐type microglia by immunofluorescent double‐staining of microglia markers Iba1/CD86 and Iba1/CD206, respectively. We found that microglia converted to M1 type after chronic sleep deprivation (Figure 3a,b). There was a significant increase in the levels of fractional inflammatory factors IL‐6 and TNF‐α (Figure 3c,d), and the damage was aggravated in the Inhibitor group. In addition, a study (Schulze‐Osthoff et al., 1992) demonstrated that the large amount of pro‐oxidative by‐products produced by excessive oxidative stress can also stimulate TNF‐α activity, and conversely, TNF‐α induces more harmful substances to be produced, further aggravating the oxidative stress, leading to a vicious cycle between excessive oxidative stress and neuroinflammation, which ultimately impairs cognitive functions such as learning and memory. It was found in the present study that administration of melatonin receptor agonists could convert microglia to M2 type and partially restore the levels of inflammatory factors IL‐6 and TNF‐α, counteracting the neuroinflammatory damage caused by chronic sleep deprivation.

Thus, our findings suggest that chronic sleep deprivation promotes the conversion of microglia to the M1 type and increases the release of inflammatory factors, while melatonin receptor agonists can partially counteract neuroinflammatory damage.

### 3.4 M1‐type microglia‐induced NF‐κB pathway further induced neuroinflammation and aggravated hippocampal neuronal injury and apoptosis, which was more severe in the melatonin receptor inhibitor group and significantly improved by administration of melatonin receptor agonist

NF‐κB is present in almost all animal cell types and controls many processes, including inflammation, apoptosis, immunity, cell survival, and cancer, and regulates the inducible expression of immune and inflammatory response genes (Shabab et al., 2017). IκB is an important member of the NF‐κB signaling pathway and primarily regulates NF‐κB activation and transcription. IκB is associated with two subunits of NF‐κB (p65 and p50) that are present in the cytoplasm in an inactivated state. When this pathway is activated, IκB is degraded, allowing the two subunits of NF‐κB, especially p65, to be transferred to the nucleus for activation and binding to the corresponding inflammation‐related genes, initiating inflammatory cytokine transcription and inducing inflammation.

**FIGURE 4:**
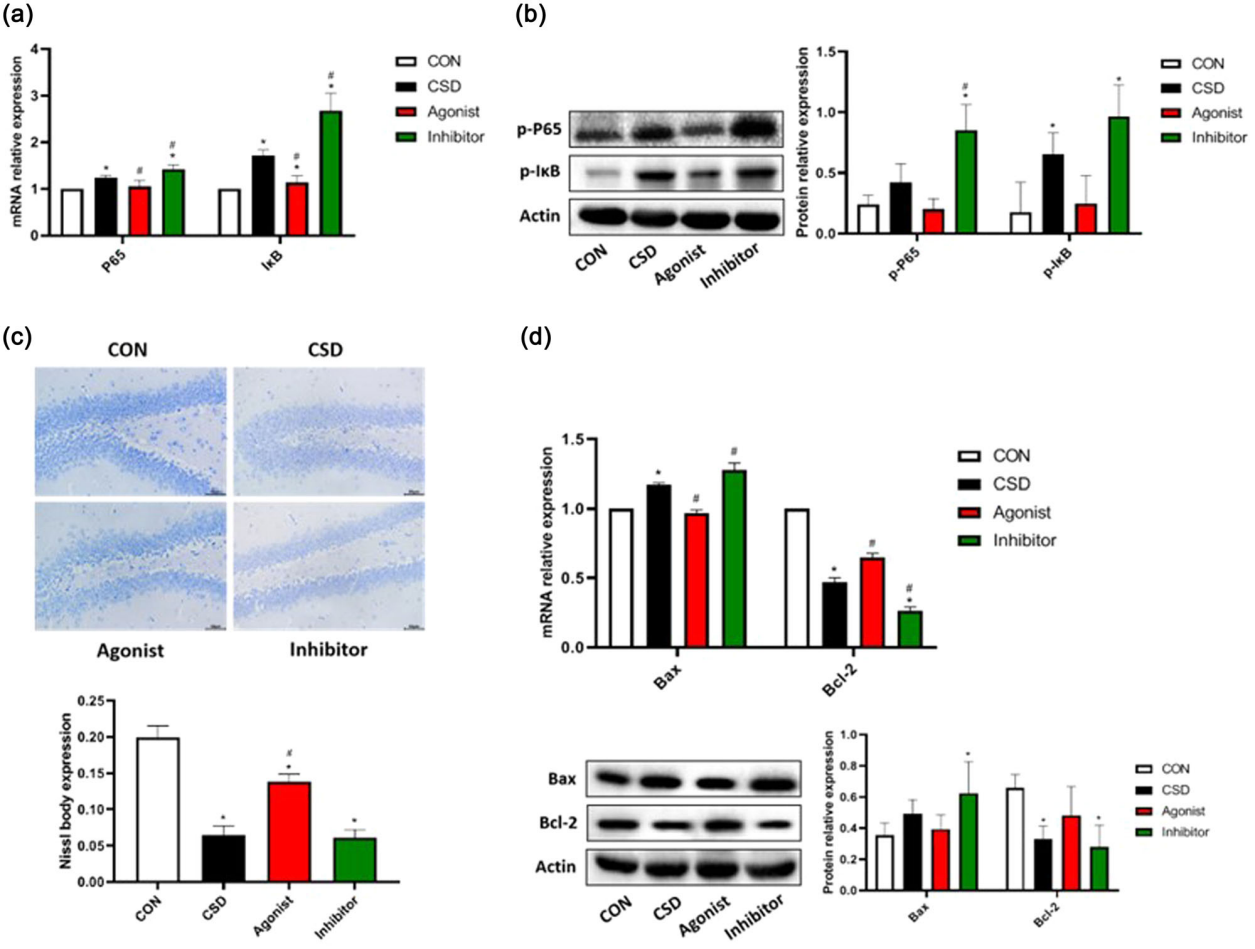
Analysis of Nissl staining and the expression of NF‐κB inflammatory pathway‐related factors. **(a)The qPCR analysis of IκB and P65**. Compared to CON, the mRNA level of P65 and IκB was greatly elevated in the CSD group (P ＜ 0.05). Compared to the CSD group, P65 and IκB were greatly downregulated in agonist group (P ＜ 0.05). On the opposite, the mRNA level of P65 and IκB was further elevated by the treatment of melatonin inhibitor (P ＜ 0.05). **(b)The Western blotting analysis of p‐IκB and p‐P65**. Compared to CON, p‐IκB was greatly upregulated in the CSD group (P ＜ 0.05). Compared to the CSD group, the protein level of p‐P65 was significantly upregulated in the inhibitor group (P ＜ 0.05). In the agonist group, the expression of p‐P65 and p‐IκB was decreased but not significantly. **(c)Analysis of Nissl staining**. The function of Nissl body is to synthesize structural proteins needed to renew organelles, enzymes needed to synthesize neurotransmitters, and peptides for neuromodulation. When neurons are damaged or overworked, Nissl bodies decrease, disintegrate, or even disappear. During recovery from injury or fatigue, Nissl bodies reappear, increase, and can reach normal levels, so Nissl bodies can be used as a marker of neuronal functional status. The number and morphology of Nissl bodies can be detected by Nissl staining to assess neuronal damage in sleep deprivation model rats. In the CON group, abundant Nissl bodies, uniform staining, clear nucleus, and regular cells arrangement were observed. However, in the CSD group, severely damaged and disorderly arranged cells, light Nissl staining, and sparse Nissl bodies were observed. Compared to the CSD group, the number of Nissl body was greatly increased and the cell damage was greatly alleviated by the treatment of melatonin agonist, while more disorderly arranged Nissl bodies and reduced the number of Nissl body were observed in the inhibitor group. **(d)The qPCR analysis and the Western blotting analysis of Bax and Bcl‐2**. Compared to CON group, the mRNA level of Bax was greatly elevated, while the mRNA and protein levels of Bcl‐2 dramatically declined in the CSD group (P ＜ 0.05). Compared to the CSD group, the mRNA level of Bax was greatly downregulated, while Bcl‐2 was upregulated by the treatment of melatonin agonist (P ＜ 0.05) On the opposite, the mRNA level of Bax was further elevated, while the level of Bcl‐2 was further declined by the treatment of melatonin inhibitor (P ＜ 0.05). **Note**: * indicates a highly significant difference (P ＜ 0.05) between the normal control group; #indicates a significant difference (P ＜ 0.05) between the model group

NF‐κB family transcription factors are involved in the regulation of microglia M1/M2 polarization and neurodegenerative diseases (Zhao et al., 2019). As previously described, we found that chronic sleep deprivation promotes microglia conversion to the M1 type, which may induce the NF‐κB pathway (Tang & Le, 2016). We, therefore, quantified the expression of NF‐κB pathway‐associated I‐κB, P65, and their phosphorylation status. Compared with the CON and the Agonist groups, the mRNA expression of IκB and P65 was significantly upregulated in the CSD and the Inhibitor groups (Figure 4a), and the protein expression of I‐κB, P65, p‐IκB, and p‐P65 was likewise significantly upregulated (Figure 4b), indicating that NF‐κB pathway was indeed activated in the CSD group. In addition, the secretion of inflammatory cytokines TNF‐α and IL‐6 is also regulated by the NF‐κB signaling pathway, which is a downstream product of this pathway, which can be explained by the fact that the increased levels of inflammatory factors TNF‐α and IL‐6 may be secreted by microglia directly or indirectly through activation of the NF‐κB pathway after M1‐type polarization of the microglia.

Neuroinflammation is often considered a double‐edged sword, which in some cases is indeed beneficial in removing some toxic proteins and cellular debris, as well as stimulating the secretion of neurotrophic factors to promote neural repair, but excessive accumulation of inflammatory factors and release of various neurotoxic mediators induce neurotoxicity and lead to increased neuronal damage (Tang & Le, 2016). As shown in Figure 4c, the neuronal cells were severely damaged and disorganized after chronic sleep deprivation, with sparse Nissl body, and the number of Nissl body was reduced and more disorganized in the Inhibitor group. In addition, microglia activation can attack healthy neurons through phagocytosis or secreted apoptotic factors. Among the apoptosis‐related cytokines, Bax has a pro‐apoptotic effect and Bcl‐2 is an anti‐apoptotic protein that prevents the activation of Bax, and the balance between the two determines whether the cells will respond to apoptotic factors. In the present study, by observing an increase in the level of Bax and a decrease in the level of Bcl‐2 in the CSD group (Figure 4d), we hypothesized that neuronal apoptosis may have occurred in the hippocampus of the CSD rats. The NF‐κB pathway was hyperactivated after administration of melatonin inhibitors, causing the induced neuroinflammation, neuronal damage, and apoptosis more severe, while the melatonin receptor agonist group was significantly improved.

Therefore, our data show that microglia M1 type‐induced NF‐κB pathway, which further induced neuroinflammation and exacerbated the hippocampal neuronal damage and apoptosis, was more pronounced in the Inhibitor group and was partially ameliorated by the administration of melatonin receptor agonists.

## 4 DISCUSSION

It is widely accepted that sleep deprivation has a detrimental impact on cognitive functions (Jan et al., 2010; Musiek et al., 2013). Early studies on neurological stromal damage due to sleep deprivation were mostly on neurons, but in recent years, more and more studies have verified the role of neuroglia, such as astrocytes, but less on microglia (Frank & Heller, 2019; Garofalo et al., 2020). Microglia are distributed in the central nervous system of the brain and are a class of immune cells that have important roles in the perception of CNS homeostasis and neurodegenerative diseases (Inoue & Tsuda, 2018; Schwabe et al., 2020). So, how do microglia regulate sleep?

It has been found that the daily dynamics of ceramide in the brain are highly correlated with sleep–wake behavior, and this correlation is eliminated in microglia‐depleted mice. Ceramide preferentially affects microglia in the thalamic reticular nucleus (TRN), and normal wakefulness is compromised when TRN microglia are depleted. Anterior TRN activity is inhibited in normal mice, whereas in microglia‐depleted mice, hyperactivation of anterior TRN reverses impaired waking behavior; thus, it is hypothesized that stable wakefulness can be restored in mice by either activating the anterior TRN neurons or inhibiting ceramide production (Liu et al., 2021).

It has been shown that microglia are considered to be the main source of IL1‐β and TNF‐α in the central nervous system (Ingiosi et al., 2013), and this experiment demonstrated that sleep deprivation increased the protein levels of the pro‐inflammatory cytokines TNF‐α, IL1‐β, IL‐6, and IL‐8 and decreased the protein levels of the anti‐inflammatory cytokines IL‐4 and IL‐10 in the rat hippocampus, which is also consistent with the findings of the previous studies (Manchanda et al., 2018; Wadhwa et al., 2017; Zielinski et al., 2014). Microglia are induced into M1 type under stressful conditions and release large amounts of inflammatory factors causing neuroinflammatory responses, and then converted to M2 type after the inflammation subsides, which plays an important role in neuroprotection (Dowlati et al., 2010). Therefore, M1/M2 polarization of microglia has an important role in the generation of neuroinflammation in the central nervous system, which activates inflammatory signaling pathways and aggravates the disease process (De Pablos et al., 2014). Similarly, our study found that chronic sleep deprivation induces excessive oxidative stress activated microglial M1 polarization, which increases the release of inflammatory molecules. Moreover, M1‐type microglia also activate the NF‐κB pathway, further inducing neuroinflammation and exacerbating hippocampal neuronal cell damage and apoptosis.

Melatonin has been identified to interact with clock genes to regulate circadian rhythms and participate in the regulation of immune and inflammatory cell functions. Melatonin also exerts significant direct or indirect antioxidant and anti‐inflammatory effects such as scavenging oxygen free radicals and inhibiting lipid peroxidation. Melatonin is reported to improve sleep. However, the role of melatonin in sleep‐related cognition remains controversial. We found that chronic sleep deprivation induces injuries in the intermediate‐term and long‐term hippocampal‐dependent spatial learning and working memory, which are in line with most previous studies (Alzoubi et al., 2016, 2019). Our data suggested that chronic sleep deprivation‐induced cognitive impairment could be ameliorated by melatonin agonists, which instead were aggravated by melatonin receptor inhibitors. It may thus be demonstrated that melatonin could ameliorate chronic sleep deprivation related cognitive impairment.

It was previously found that deletion of BMAL1 leads to the excessive prooxidant and proinflammatory phenotype (Early et al., 2018; Xie et al., 2020). Our data demonstrated that chronic sleep deprivation resulted in the BMAL1 downregulation, but BMAL1 was upregulated with melatonin receptor agonists, which in turn inhibited excessive oxidative stress in the hippocampus, thereby polarizing the microglia M2 phenotype and alleviating the inflammatory response in chronic sleep‐deprived rats. The opposite result was observed using melatonin receptor inhibitors. We also found that activation of microglial M1 polarization, which could also be additive to apoptosis, was another cause of cognitive impairment. Apoptosis was similarly reduced in chronic sleep‐deprived rats treated with melatonin receptor agonists, which may be related to the inhibition of microglial activation. Thus, inhibition of microglia activation may provide a potential treatment for stress‐induced cognitive impairment.

## 5 CONCLUSION

The results of this study showed that the expression of inflammatory factors TNF‐α and IL‐6, oxidative stress factors SOD and MDA, apoptosis‐related genes Bax, Bcl‐2, as well as NF‐κB inflammatory pathway‐related proteins P65 and IκB could be activated in sleep‐deprived rats, and induced the occurrence of hippocampal neuronal cells polarization to M1‐type microglia, indicating that chronic sleep deprivation is closely related to neuroinflammation and apoptosis. The activation of microglia culminated in the release of inflammatory cytokines, enhanced neuroinflammation, and cognitive impairment during stress and other pathological conditions. After administration of melatonin receptor agonist treatment to rats, the expression level of BMAL1 was upregulated, the responses of inflammation, apoptosis, and oxidative stress were effectively improved, microglia were converted to M2 type, and the cognitive behavior of rats was somewhat improved. In contrast, the Inhibitor group exacerbated the above response process, further suggesting the important role of melatonin in the mechanism of chronic insomnia with anti‐inflammatory and anti‐apoptotic effects.

Collectively, we speculated that melatonin upregulated BMAL1 to attenuate chronic sleep deprivation‐related cognitive impairment by alleviating oxidative stress.

## AUTHOR CONTRIBUTIONS

Guoshuai Yang contributed to the conception and design of the study. Yujie Hu and Jierong Yin performed the experiments. Yujie Hu and Jierong Yin organized the database. Yujie Hu and Jierong Yin performed the statistical analysis. Yujie Hu wrote the first draft of the manuscript. Jierong Yin wrote the sections of the manuscript. All authors contributed to the manuscript revision, read, and approved the submitted version.

## CONFLICT OF INTEREST

The authors declare no conflict of interest.

### PEER REVIEW

The peer review history for this article is available at: https://publons.com/publon/10.1002/brb3.2836

## Data Availability

The data that support the findings of this study are available from the corresponding author upon reasonable request.
